# Demographic analysis of arrhenotokous parthenogenesis and bisexual reproduction of Frankliniella occidentalis (Pergande) (Thysanoptera: Thripidae)

**DOI:** 10.1038/s41598-018-21689-z

**Published:** 2018-02-20

**Authors:** Tianbo Ding, Hsin Chi, Ayhan Gökçe, Yulin Gao, Bin Zhang

**Affiliations:** 1 Key Lab of Integrated Crop Pest Management of Shandong, College of Plant Health and Medicine, Qingdao Agricultural University, Qingdao, 266109 P. R. China; 2 Department of Plant Production and Technologies, Faculty of Agricultural Sciences and Technologies, Niğde Ömer Halisdemir University, Niğde, 51240 Turkey; 3 State Key Laboratory for Biology of Plant Diseases and Insect Pests, Institute of Plant Protection, Chinese Academy of Agricultural Sciences, Beijing, 100193 P. R. China

## Abstract

*Frankliniella occidentalis* (Pergande) (Thysanoptera: Thripidae) is a serious pest that is capable of bisexual and arrhenotokous reproduction. In arrhenotokous reproduction, virgin females initially produce male offspring; later, when their sons are sexually mature, the mothers begin bisexual reproduction by carrying out oedipal mating with their sons. Because a virgin female produces many male offspring before oedipal mating occurs, multiple oedipal mating is common. In this study, we investigated the effect of multiple oedipal mating on the population growth of *F. occidentalis* by using the age-stage, two-sex life table theory. In the arrhenotokous cohorts, all unfertilized eggs developed into males. In the bisexual cohorts, the offspring sex ratio was significantly female biased with the mean number of female offspring and male offspring being 72.68 and 29.00, respectively. These were the same as the net reproductive rate of female offspring and male offspring. In arrhenotokous cohorts, the number of males available for oedipal mating significantly affected the production of female offspring. The number of female offspring increased as the number of sons available for oedipal mating increased. Correctly characterizing this unique type of reproduction will provide important information for predicting the timing of future outbreaks of *F. occidentalis*, as well as aiding in formulating successful management strategies against the species.

## Introduction

The western flower thrips (WFT), *Frankliniella occidentalis* (Pergande) (Thysanoptera: Thripidae), is one of the most economically important insect pests of many horticultural crops especially in greenhouses^1,2^. The WFT causes damage to plants not only via direct feeding on leaves or flowers, but also by transmitting many plant viruses^3–5^. This pest, originally native to North America^6^, has spread to more than 60 countries since the late 1970’s, including Canada, Australia, United Kingdom, and Japan^7^. In China, an established WFT population was found in Beijing in 2003^8^ and has since been detected in a number of other provinces^9–13^.

The WFT has a haplo-diploid breeding system, with females developing from fertilized eggs and males from unfertilized eggs^14^. Most females produce female offspring only after mating with males; although Kumm and Moritz observed that a tiny fraction of unfertilized eggs developed into females^15^. Virgin females can, however, mate with their sons and establish bisexual population by arrhenotokous reproduction^16^. This type of reproduction contributes to a high survival probability in a population, in conjunction with the rapid expression and development of genes including resistance associated genes^17,18^. A number of WFT life tables and population parameters have been published using the traditional female age-specific life table theory^19–22^. Unfortunately, because, female age-specific life tables, in the sense of Lewis^23^, Leslie^24^, Birch^25^, and Carey^26^, inherently overlook the male population and are incapable of describing the stage differentiation, their applications are limited and will inevitably result in errors in data analysis and population projection^27^.

To integrate the effect of sex ratio, the contribution made by male individuals, and the stage differentiation, Chi and Liu^28^ and Chi^29^ developed the age-stage, two-sex life table. By using the age-stage, two-sex life table, Tuan *et al*. developed a novel procedure for the demographic analysis of arrhenotokous parthenogenesis in the spider mite, *Tetranychus urticae* Koch (Acari: Tetranychidae)^30^. In this study, we collected life table data for WFT with bisexual and arrhenotokous reproduction using a variable number of males for oedipal mating. We then analyzed the data using the age-stage, two-sex life table technique to quantitatively assess and compare the effects that arrhenotokous reproduction and variations in the number males available for oedipal mating have on demographic parameters.

## Materials and Methods

### Insect Rearing and Host Plant

The WFTs were originally collected from clover plants, *Trifolium repens* L. (Fabales: Fabaceae), at the Experimental Station of Qingdao Agricultural University (N 36°31′, E 120°39′) in 2008^9^. Using morphological characters, the WFT was provisionally identified according to Mound and Kibby (1998)^31^; we then used molecular tools (DNA barcoding) to identify the tested thrips according to the BLAST result^32^. The PCR primers (LCO1490: 5′-GGTCAACAAATCATAAAGATATTGG-3′ and HCO2198: 5′-TAAACTTCAGGGTGACCAAAAAATCA-3′) were used to amplify mitochondrial cytochrome c oxidase subunit I (COI) of the thrips species^33^. Voucher specimens have been deposited in the Specimen Room of Qingdao Agricultural University. The purple cabbage, *Brassica oleracea* L. (Brassicales: Brassicaceae), was used as the host plant for rearing WFT in growth chambers (Shanghai Yiheng Instruments, China) set at 25 ± 1 °C, 55 ± 5% relative humidity (RH), and a photoperiod of 16: 8 (L: D) h. WFTs were reared under these conditions in excess of 300 generations prior to the life table study.

### Life Table Study

In the life table studies, WFTs were individually reared on leaf discs (1.0 cm in diameter) cut from purple cabbage leaves and placed in 1.5-mL centrifuge tubes (Shanghai Sangon Biotech, China). A triangular piece (1.5 cm in height and 0.5 cm in base) of premoistened filter paper was placed in the tube to prevent desiccation of the leaf disc. A 0.8 cm diam. hole was punched in the center of the lid and covered with 200-mesh nylon gauze for ventilation. The lid edges were sealed with Parafilm (PM-996, Bemis Company, Oshkosh, WI, USA) to prevent the WFTs from escaping. Because the WFT eggs were laid inside the plants tissues, it was impractical to observe their development in order to precisely determine this demographic parameter. Instead, we based the life table using individuals surviving to the adult stage for both the parental and offspring generations^34^. All studies were carried out in growth chambers set at 25 ± 1 °C, 55 ± 5% relative humidity (RH), and a photoperiod of 16: 8 (L: D) h.

### Life Table of Bisexual WFT

Fifty pairs of WFT were randomly selected and each pair placed on separate leaf discs in 15-mL plastic tubes (Shanghai Sangon Biotech, China), and allowed to mate and oviposit for 24 h. After 24 h, the adults were removed and the leaf discs were observed daily for emergence of the larvae. A total of 51 newly hatched larvae were used for the life table study. Each larva was individually placed in a 1.5-mL centrifuge tube with one purple cabbage leaf disc as described above. The development stage and survival data were recorded daily, and the leaf discs were replaced with new discs every 2 days. When adults emerged, a single virgin male and female WFT were paired in a new 1.5-mL centrifuge tube. The cabbage discs were removed to collect the eggs, and replaced with a fresh disc daily. The daily fecundity and survival of adults were recorded until the death of all individuals. If an individual died earlier than its mate, it was replaced with another individual of the same sex from the mass-rearing colony. Individuals supplemented from the mass-rearing colony were for mating purposes only and were excluded from data analysis. To assess the sex ratio of offspring laid by the female WFTs at different ages, eggs were kept separated until developing to the adult stage to allow for sex determination.

### Life Table of Arrhenotokous WFT

Over five hundred newly hatched larvae were collected and individually reared in 1.5-mL centrifuge tubes containing one purple cabbage leaf disc. After the emergence of adults, virgin females were moved to individual 1.5-mL centrifuges tube for the life table study. The survival of the females was recorded and leaf disk replaced daily. Leaf disks with eggs were kept until the emergence of larvae. Each newly emerged larva was transferred to a new centrifuge tube containing a new leaf disc and observed daily until reaching the adult stage. The first few male offspring to emerge as adults were transferred back to the tub containing its mother. To study the effects that variable male ratios may have on arrhenotokous reproduction, eight oedipal mating sex-ratios (mother: son) were used, i.e., 1 F:0 M, 1 F:1 M, 1 F:2 M, 1 F:3 M, 1 F:4 M, 1 F:5 M, 1 F:6 M, 1 F:nM. In the 1 F:0 M treatment, no male was transferred back to mate with its mother. In the 1 F:1 M, 1 F:2 M, 1 F:3 M, 1 F:4 M, 1 F:5 M, 1 F:6 M treatments, constant sex ratios were maintained throughout the experiment. The numbers of virgin females used were 36, 38, 36, 35, 37, 30, 35, and 45 individuals in treatments 1 F:0 M, 1 F:1 M, 1 F:2 M, 1 F:3 M, 1 F:4 M, 1 F:5 M, 1 F:6 M, 1 F:nM, respectively. The survival and daily fecundity were recorded until the death of the mother; if one or more male offspring died before its mother, new adult male offspring from the same female were substituted to maintain the sex ratios. In the 1 F:nM treatment, the number of oedipal mating males was not a constant and all male offspring adults were kept with their mother until the parental female adult died; the total number of males varied from 10 to 60. All life table studies initially began with 30–45 virgin females. Eggs produced by females after oedipal mating were kept separately and reared to the adult stage to determine the sex of the offspring. According to the procedure suggested by Huang and Chi^35^ and Mou *et al*.^34^, we included only data from the eggs that successfully developed through to the adult stage in the life table analyses.

### Life Table Analysis

The raw data were analyzed using the age-stage, two-sex life table theory^28,29,35^. The age-stage-specific survival rate (*s*_*xj*_, where *x* = age and *j* = stage), female age-stage-specific fecundity (*f*_*xj*_), age-specific survival rate (*l*_*x*_), age-specific fecundity (*m*_*x*_), age-specific maternity (*l*_*x*_*m*_*x*_), age-stage specific life expectancy (*e*_*xj*_), and the age-stage reproductive value (*v*_*xj*_) were calculated using the daily life history raw data. The life table parameters, i.e., the intrinsic rate of increase (*r*), net reproduction rate (*R*_0_), finite rate of increase (*λ*), mean generation time (*T*), adult preoviposition period (APOP), total preoviposition period (TPOP), and the fecundity of females were calculated accordingly. In the age-stage, two-sex life table, the age-specific survival rate (*l*_*x*_) is calculated as follows^28^:

1 $${l}_{x}=\sum _{j=1}^{k}{s}_{xj}$$

where *k* is the number of stages. The age-specific fecundity (*m*_*x*_) is calculated as:

2 $${m}_{x}=\frac{\sum _{j=1}^{k}{s}_{xj}{f}_{xj}}{\sum _{j=1}^{k}{s}_{xj}}$$

The intrinsic rate of increase (*r*) is estimated iteratively by using the bisection method from the Euler-Lotka equation as follows:

3 $$\sum _{x=0}^{\infty }{e}^{-r(x+1)}{l}_{x}{m}_{x}={\rm{1}}$$

with age indexed from 0^36^. The net reproductive rate (*R*_0_) is calculated as follows:

4 $${R}_{{\rm{0}}}=\sum _{x=0}^{\infty }{l}_{x}{m}_{x}$$

The mean generation time (*T*) is defined as the length of time that is required by a population to increase to *R*_0_-fold its initial size at the stable age-stage distribution, and is calculated as:

5 $$T=\frac{\mathrm{ln}\,{R}_{0}}{r}$$

The age-stage life expectancy (*e*_*xj*_) is the length of time that an individual of age *x* and stage *j* is expected to survive and is calculated as:

6 $${e}_{xj}=\sum _{i=x}^{\infty }\sum _{y=j}^{k}{s^{\prime} }_{iy}$$

where ${s^{\prime} }_{iy}$ is the probability that an individual of age *x* and stage *j* will survive to age *i* and stage *y* and is calculated by assuming ${s^{\prime} }_{xj}=1$, following the procedures described in^29^ and Chi and Su^37^. According to Fisher^38^, the age-stage reproductive value (*v*_*xj*_) is defined as the contribution of an individual of age *x* and stage *j* to the future population. According to Huang and Chi^35^ and Tuan *et al*.^39,40^, the reproductive value (*v*_*xj*_) in the age-stage, two-sex life table is calculated as:

7 $${v}_{xj}=\frac{{e}^{r(x+1)}}{{s}_{xj}}\sum _{i=x}^{\infty }{e}^{-r(i+1)}\sum _{y=j}^{k}{s^{\prime} }_{iy}{f}_{iy}$$

In arrhenotokous reproduction, the offspring sex ratio varies with the age of the female. To determine the effect of offspring sex ratio on the population parameters, we also analyzed the data using the age-stage, two-sex life table method with the offspring sex ratio dependent on female age^35,41^. The net reproductive rate of the parent cohort can be calculated as:

8 $${R}_{0,total}=\sum _{x=0}^{\infty }{l}_{x,F}{m}_{x,total}=\sum _{x=0}^{\infty }{l}_{x,F}{m}_{x,F}+\sum _{x=0}^{\infty }{l}_{x,F}{m}_{x,M}+\sum _{x=0}^{\infty }{l}_{x,F}{m}_{x,N}$$

where *l*_*x,F*_ is the reproductive proportion of female parents at age *x*, while *m*_*x,F*_, *m*_*x,M*_, and *m*_*x,N*_ are the female, male, and N-type offspring produced by female parents at age *x* (N-type represents the offspring that die in the preadult stage). Because only individuals surviving to the adult stage were included, Equation 8 can be simplified as:

9 $${R}_{0,total}=\sum _{x=0}^{\infty }{l}_{x,F}{m}_{x,total}=\sum _{x=0}^{\infty }{l}_{x,F}{m}_{x,F}+\sum _{x=0}^{\infty }{l}_{x,F}{m}_{x,M}$$

According to Huang and Chi^35^, if the offspring sex ratio is dependent on mother’s age, the intrinsic rate can be estimated from the following equation:

10 $$\sum _{x=0}^{\infty }{e}^{-r(x+1)}{l}_{x,F}{m}_{x,F}=1$$

We then used the above results to calculate the stable sex ratio (F:M) accordingly. The mean generation time is calculated as:

11 $$T=\frac{\mathrm{ln}\,{R}_{0,total}}{r}$$

The raw data and population parameters were analyzed using the computer program TWOSEX-MSChart^42^, which is available at no cost at http://140.120.197.173/Ecology/Download/TWOSEX-MSChart.rar. To obtain stable estimates of variances and standard errors of the developmental time, longevity, fecundity, and population parameters, we used the bootstrap technique^27,43,44^, with 100,000 bootstraps^45^. The paired bootstrap test was used to assess differences among treatments^44^. The figures were prepared using SigmaPlot, version 12.0 (Systat Software Inc.).

### Compliance with ethical standards

This research did not involve any human participants and/or animals, other than the western flower thrips, *F. occidentalis*. Informed Consent: Not applicable.

## Results

### Preadult development, APOP and TPOP

Significant differences were observed in the preadult developmental times and adult longevity between females and males in the bisexual cohort of WFT. Among all arrhenotokous cohorts, the female adults in the 1 F:0 M cohort (no oedipal mating) lived significantly longer than females in other cohorts, while the longevity of female adults in the 1 F:nM cohort (unlimited oedipal mating) was the shortest in all treatments (Table 1). The female adult longevity in the bisexual cohort was significantly shorter than in all arrhenotokous cohorts.

**Table 1:** Preadult duration, adult longevity, total fecundity and proportion of female offspring of *Frankliniella occidentalis* in different treatments of arrhenotokous and bisexual reproduction.

Cohort	Sex	*n*	Preadult duration	Adult longevity	Fecundity (Male and female offspring)	Proportion of female offspring
1 F:0 M	F	36	12.11 ± 0.09A	30.83 ± 1.00A	115.44 ± 2.61B	0 G
1 F:1 M	F	38	11.74 ± 0.10BC	23.00 ± 0.57B	91.97 ± 3.07D	7.44 ± 0.81%F
1 F:2 M	F	36	11.69 ± 0.12BC	22.00 ± 0.62B	90.89 ± 3.88D	11.46 ± 1.12%E
1 F:3 M	F	35	11.71 ± 0.12BC	21.83 ± 0.59B	92.57 ± 4.58D	13.95 ± 1.08%E
1 F:4 M	F	37	11.57 ± 0.12C	22.89 ± 0.49B	90.86 ± 5.09D	20.70 ± 1.42%D
1 F:5 M	F	30	11.50 ± 0.09C	22.57 ± 0.63B	96.50 ± 6.31D	25.15 ± 1.66%BC
1 F:6 M	F	35	12.00 ± 0.13AB	21.86 ± 0.36B	129.34 ± 3.03A	24.61 ± 1.43%CD
1 F:nM	F	45	11.71 ± 0.10BC	19.09 ± 0.42C	118.53 ± 3.67B	26.15 ± 1.03%B
Bisexual	F	38	11.74 ± 0.10BC	17.82 ± 0.64D	101.68 ± 4.04C	71.48 ± 0.29%A
	M	14	10.79 ± 0.18D	9.78 ± 0.37E		

Means in the same column followed by the same upper case letter denotes no significant difference between different cohorts.

In all arrhenotokous reproduction treatments, the APOP’s and TPOP’s for producing male offspring were significantly shorter than those for producing female offspring (Table 2). However, no significant differences were found in the APOP and TPOP for producing male and female offspring in bisexual reproduction. The APOP and TPOP for producing female offspring were significantly shorter in bisexual than those found in arrhenotokous reproduction with oedipal mating (Table 2). In arrhenotokous reproductions, significant differences in the APOP’s and TPOP’s for producing female offspring were observed among different oedipal mating sex ratios; the APOP and TPOP for producing female offspring decreased with increased numbers of oedipal mating males. The APOP and TPOP values for producing male offspring were significantly shorter in bisexual than in arrhenotokous reproduction (Table 2). No significant difference in the APOP for producing male offspring was found in oedipal mating reproduction involving a constant number of males. In contrast to the TPOP for producing female offspring, fewer significant differences in the TPOP for producing male offspring were found among different oedipal mating treatments (Table 2).

**Table 2:** APOP, TPOP, oviposition days, and fecundity of *Frankliniella occidentalis* producing male and female offspring.

	*n*	Cohort	APOP	TPOP	Ovi. days	Fecundity (male offspring)
Production of male offspring	36	1 F:0 M	2.50 ± 0.08C	14.61 ± 0.09AB	24.95 ± 0.73A	115.44 ± 2.61A
	38	1 F:1 M	2.84 ± 0.11Ab	14.58 ± 0.13BCb	16.11 ± 0.47Ca	85.13 ± 2.56Ca
	36	1 F:2 M	2.78 ± 0.14Ab	14.47 ± 0.21BCb	16.19 ± 0.60Ca	80.47 ± 3.41CDa
	35	1 F:3 M	2.86 ± 0.06Ab	14.57 ± 0.12BCb	16.54 ± 0.65Ca	79.66 ± 3.11CDa
	37	1 F:4 M	2.73 ± 0.07Ab	14.30 ± 0.12Cb	15.95 ± 0.66Ca	72.05 ± 4.53Da
	30	1 F:5 M	2.83 ± 0.07Ab	14.33 ± 0.09Cb	16.77 ± 0.66Ca	72.23 ± 3.62Da
	35	1 F:6 M	2.83 ± 0.10Ab	14.83 ± 0.11Ab	18.80 ± 0.37Ba	97.51 ± 2.34Ba
	45	1 F:nM	2.64 ± 0.09Bb	14.36 ± 0.14BCb	14.02 ± 0.41Da	87.53 ± 2.79Ca
	38	Bisexual	1.82 ± 0.06Da	13.55 ± 0.12Da	13.61 ± 0.50Da	29.00 ± 1.20Eb
	***n***	**Cohort**	**APOP**	**TPOP**	**Ovi. days**	**Fecundity (female offspring)**
Production of female offspring	36	1 F:0 M	—	—	—	—
	38	1 F:1 M	14.82 ± 0.31Aa	26.55 ± 0.32Aa	4.34 ± 0.53Fb	6.84 ± 0.88Fb
	36	1 F:2 M	14.25 ± 0.24ABa	25.94 ± 0.31ABa	5.50 ± 0.42EFb	10.42 ± 1.18Eb
	35	1 F:3 M	14.06 ± 0.12Ba	25.77 ± 0.17Ba	6.57 ± 0.66Deb	12.91 ± 1.59Eb
	37	1 F:4 M	13.46 ± 0.17Ca	25.03 ± 0.21Ca	7.73 ± 0.41CDb	18.81 ± 1.45Db
	30	1 F:5 M	12.80 ± 0.37CDa	24.30 ± 0.42CDa	8.80 ± 0.70BCb	24.27 ± 2.99CDb
	35	1 F:6 M	11.63 ± 0.25Da	23.63 ± 0.26Da	10.03 ± 0.39Bb	31.83 ± 2.24BCb
	45	1 F:nM	10.96 ± 0.09Ea	22.67 ± 0.15Ea	8.13 ± 0.46Cb	31.00 ± 1.76Bb
	38	Bisexual	1.76 ± 0.07Fa	13.50 ± 0.12Fa	14.97 ± 0.59Aa	72.68 ± 2.89Aa

Means in the same column followed by the same upper case letter denotes no significant difference between different cohorts, while means followed by the same lower case letter denotes no significant difference between production of male and female offspring in the same treatments.

### Age-stage survival rate and fecundity

**Figure 1:**
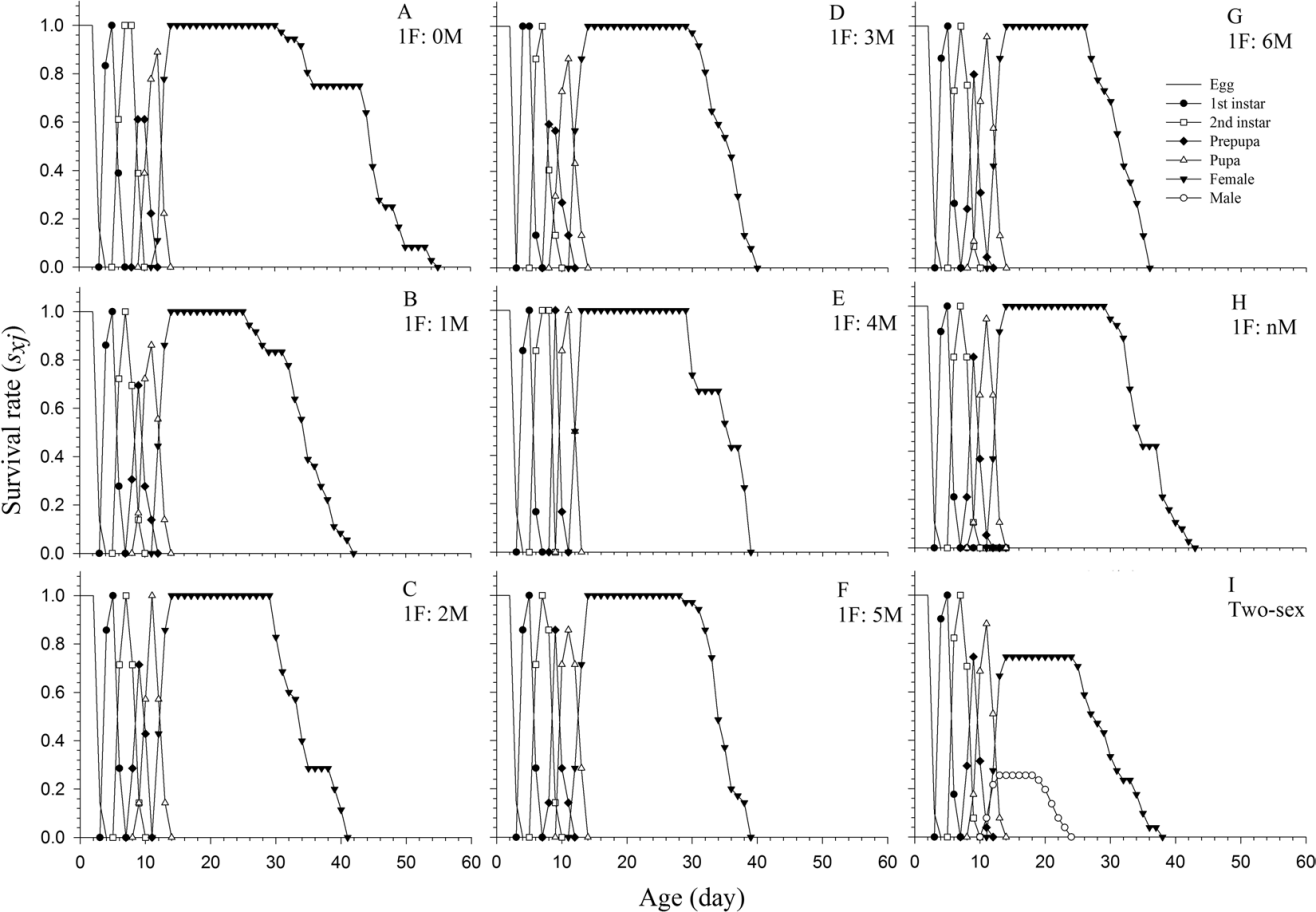
Age-stage survival rate (*s*_*xj*_) of *Frankliniella occidentalis* at different arrhenotokous sex ratios and bisexual population. A = 1 F:0 M, B = 1 F:1 M, C = 1 F:2 M, D = 1 F:3 M, E = 1 F:4 M, F = 1 F:5 M, G = 1 F:6 M, H = 1 F:nM, I = Two-sex.

**Figure 2:**
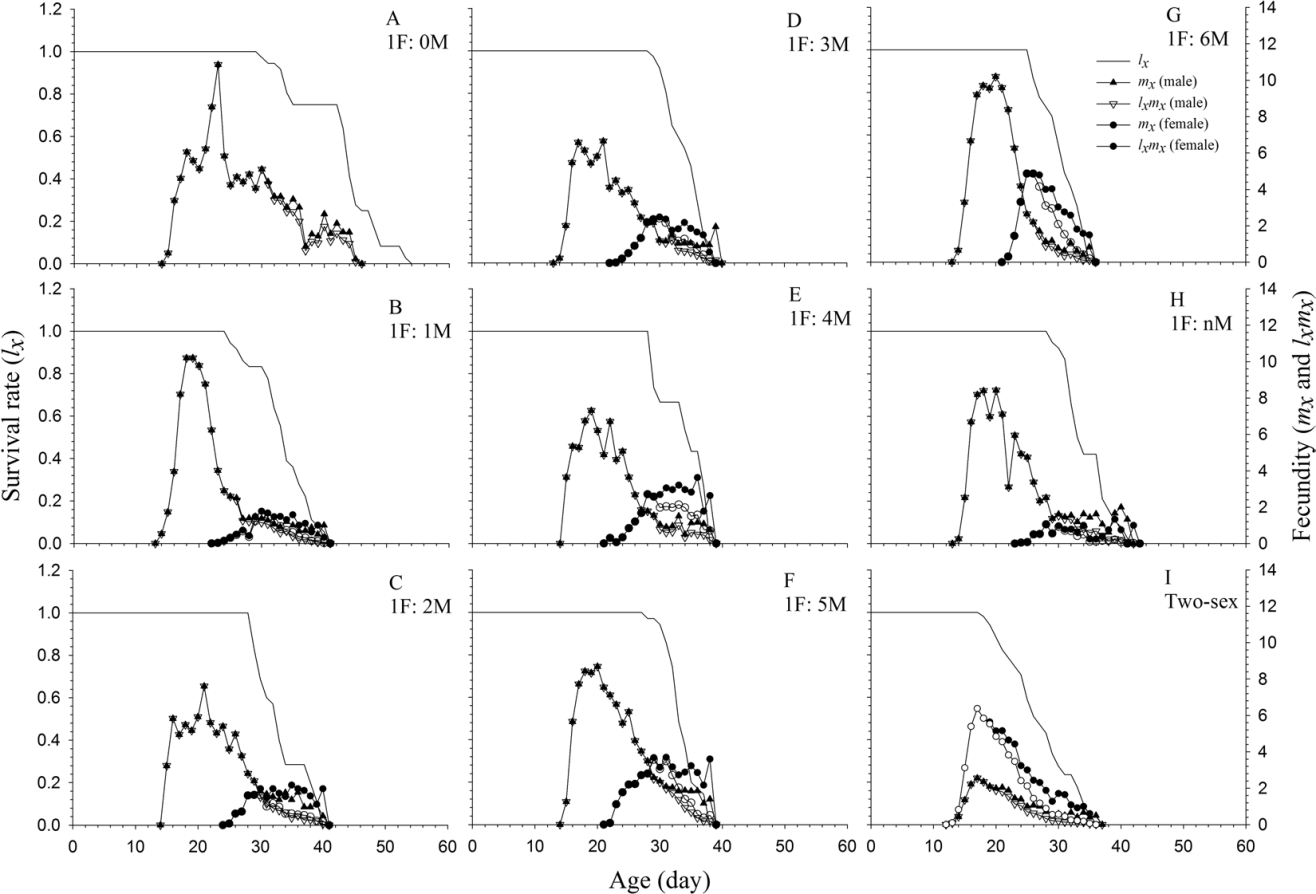
Age-specific survival rate (*l*_*x*_), fecundity (*m*_*x*_) and maternity (*l*_*x*_*m*_*x*_) o of *Frankliniella occidentalis* at different arrhenotokous sex ratios and bisexual population. A = 1 F:0 M, B = 1 F:1 M, C = 1 F:2 M, D = 1 F:3 M, E = 1 F:4 M, F = 1 F:5 M, G = 1 F:6 M, H = 1 F:nM, I = Two-sex.

Detailed development and stage differentiation of all cohorts can be observed in Fig. 1. Clear overlapping in the age-stage survival curves (*s*_*xj*_) was found in successive stages, demonstrating the variable developmental rates that occurred in WFT individuals of bisexual and arrhenotokous reproductive types (Fig. 1). Only female curves were present in the arrhenotokous cohorts (Fig. 1A–H), but two separate curves for the female and male adults were obvious in the bisexual cohort (Fig. 1I). The WFT age-specific survival rate (*l*_*x*_), fecundity (*m*_*x*_), and net maternity (*l*_*x*_*m*_*x*_) are plotted in Fig. 2. In arrhenotokous reproduction with oedipal mating, the fecundity curve of the male offspring was variable among different oedipal mating sex ratios, with the female offspring beginning to emerge from age 21 to 24 d (Fig. 2B–H). Without oedipal mating, WFT females produced only male offspring (Fig. 2A). In bisexual cohorts, females WFT began to produce both male and female offspring at age 13 d, with the daily fecundity when producing female offspring higher than that when producing male offspring throughout the entire oviposition period (Fig. 2H). In arrhenotokous reproduction, as the number of oedipal mating males increased, the number and proportion of female offspring also increased (Fig. 2B–H). In the bisexual cohort, no differences were observed between the number of oviposition days needed for producing female offspring and for producing male offspring. The number of oviposition days needed for producing female offspring in the bisexual cohort was, however, significantly higher than in all of the arrhenotokous cohorts (Table 2). In the arrhenotokous cohorts with constant numbers of oedipal mating males, the number of oviposition days needed for producing females increased with increases in the number of males used for oedipal mating, except in the 1 F:4 M and 1 F:5 M sex ratios, where no significant differences were found. The number of oviposition days needed for producing male in the arrhenotokous cohort without oedipal mating was significant longer than in other cohorts. The number of oviposition days needed for producing male offspring in the bisexual cohort was not significantly different from that of the arrhenotokous cohorts with continuous oedipal mating, but was, however, significantly shorter than in those cohorts with constant oedipal mating males.

The total fecundity (including female and male offspring) varied among treatments. However, the proportion of female offspring did show an increasing trend with the increase of oedipal males, with the highest proportion found in the bisexual cohort (Table 1). In the bisexual cohort, females produced an average of 72.68 female and 29.0 male eggs during their lifetime, while arrhenotokous females produced fewer female offspring than male offspring (Table 1). In the arrhenotokous cohorts, virgin female adults initially produced only male eggs, but after oedipal mating, they produced mainly female offspring (Fig. 2). Both the quantity and proportion of female offspring increased with increases in the number of oedipal mating males in the arrhenotokous reproduction groups (Table 1). Without oedipal mating, WFT produced an average of 115.44 male offspring, which was significantly higher than in all other treatments. The proportion of female offspring increased with the number of oedipal mating males.

### Life expectancy and reproductive value

**Figure 3:**
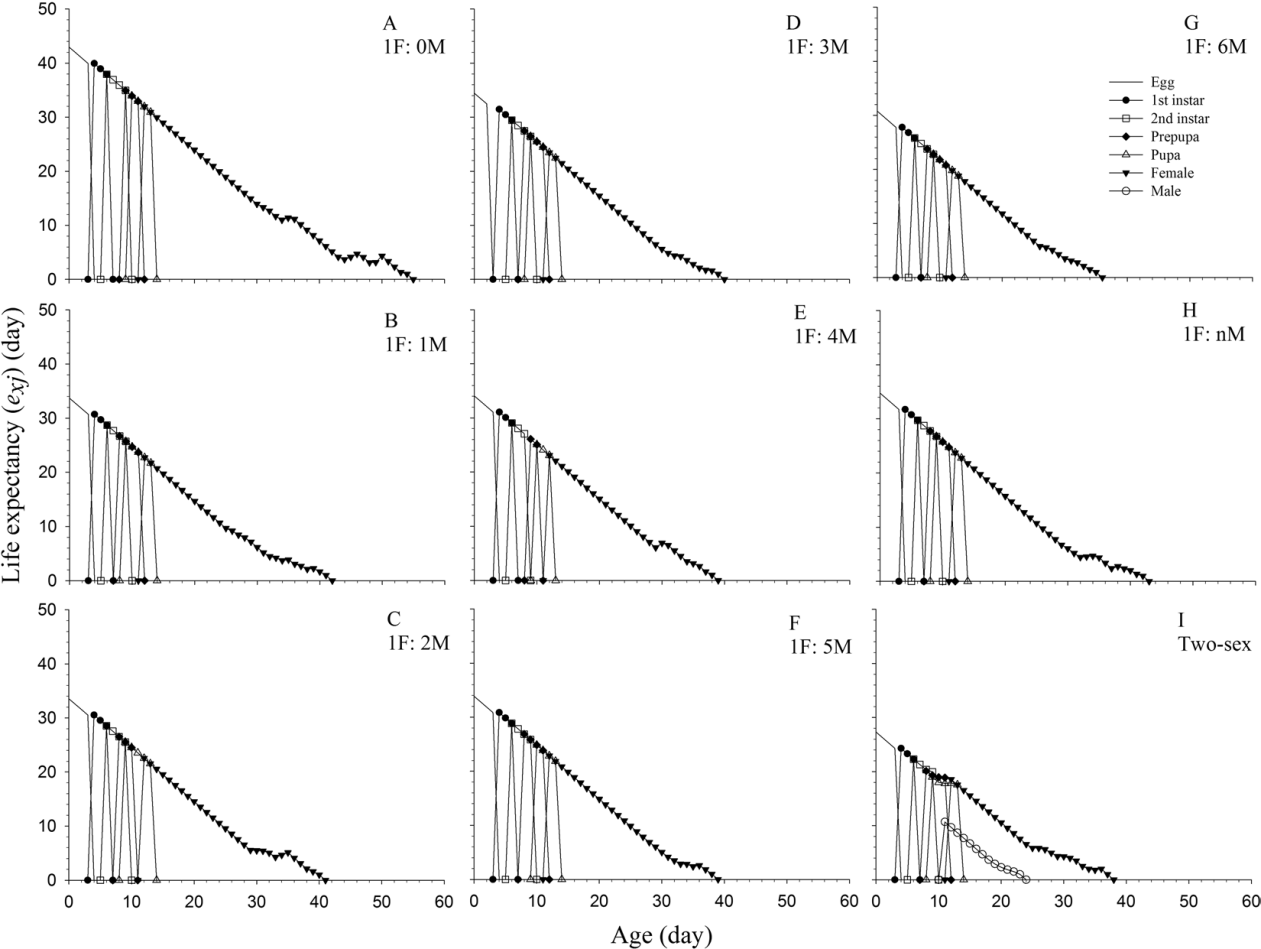
Age-stage life expectancy (*e*_*xj*_) of *Frankliniella occidentalis* at different arrhenotokous sex ratios and bisexual population. A = 1 F:0 M, B = 1 F:1 M, C = 1 F:2 M, D = 1 F:3 M, E = 1 F:4 M, F = 1 F:5 M, G = 1 F:6 M, H = 1 F:nM, I = Two-sex.

**Figure 4:**
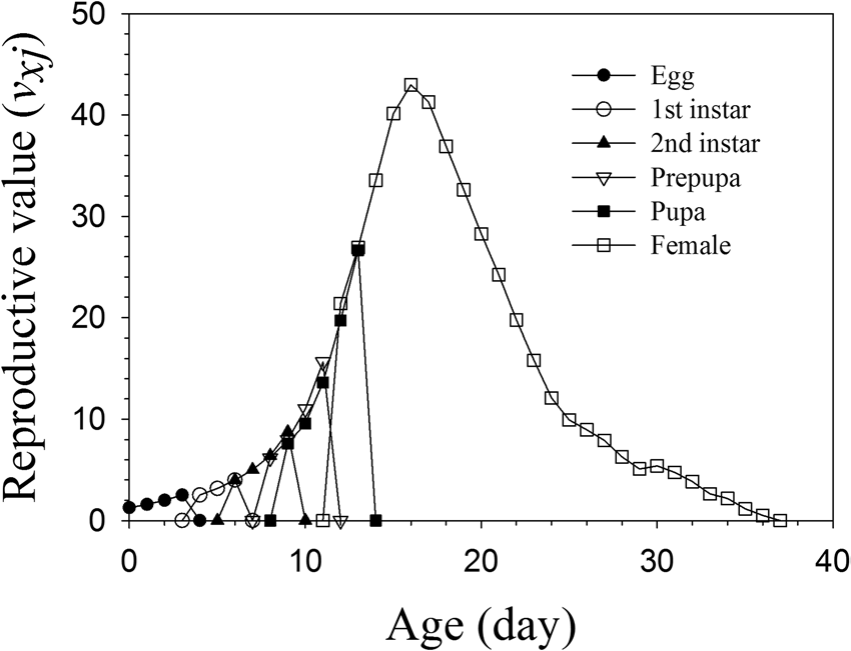
Age-stage life expectancy (*v*_*xj*_) of *Frankliniella occidentalis* in the bisexual population.

The age-stage life expectancies (*e*_*xj*_) of the bisexual and arrhenotokous cohorts of WFT are plotted in Fig. 3. The life expectancies at different ages of the bisexual cohort, were, in general, shorter than those in the arrhenotokous cohorts. As shown in Fig. 3, the *e*_*xj*_ in the arrhenotokous cohort without oedipal mating was longer than in other cohorts. The age-stage reproductive value (*v*_*xj*_) of the WFT bisexual cohort is plotted in Fig. 4. We did not plot a corresponding *v*_*xj*_ curve for the arrhenotokous cohort because calculation requires the intrinsic rate of increase, which is unavailable for this cohort.

### Population parameters

The population parameters of WFT with bisexual reproduction are listed in Table 3. Using the age-stage, two-sex life table^28,29^, values for the intrinsic rate of increase (*r*), finite rate (*λ*), net reproductive rate (*R*_0_), and mean generation time (*T*) of the bisexual cohort were 0.2295 d^−1^, 1.2579 d^−1^, 74.33 offspring, and 18.78 d, respectively. Because the offspring sex ratio is dependent on female age, we calculated the values using the method of Huang and Chi^35^; the intrinsic rate of increase (*r*), finite rate (*λ*), net reproductive rate of female offspring (*R*_0,*f*_), and mean generation time (*T*) of the female were then 0.2278 d^−1^, 1.2558 d^−1^, 72.68 offspring, and 18.82 d, respectively. Because including total eggs, i.e., male eggs that were produced before oedipal mating and female eggs produced after oedipal mating, would overestimate the population parameters, we did not use the total eggs to estimate these parameters. Similarly, because including only eggs produced after oedipal mating would underestimate the population parameters, we omitted the life table parameters of the arrhenotokous cohorts in this study.

**Table 3:** Population parameters of bisexual cohort of *Frankliniella occidentalis* (L1, the first instar; L2, the second instar).

Cohort	Age-stage, two-sex life table	Age-stage, two-sex life table with offspring sex-ratio dependent on female age
Intrinsic rate of increase (*r*) (d^−1^)	0.2295 ± 0.0052	0.2278 ± 0.0020
Finite rate of increase (*λ*) (d^−1^)	1.2579 ± 0.0066	1.2558 ± 0.0025
Net reproductive rate (*R*_0_) (offspring)	74.31 ± 6.93	*R*_0,*total*_ = 101.68 ± 4.02 *R*_0,*f*_ = 72.68 ± 2.87 *R*_0,*m*_ = 29.00 ± 1.20
Mean generation time (*T*) (day)	18.78 ± 0.16	20.29 ± 0.16
Stable stage distribution (Egg:L1:L2:Prepupa:Pupa:Adult)	0.511:0.187:0.137:0.045:0.051:0.068	0.600:0.156:0.112:0.036:0.041:0.054
Stable adult sex ratio (F:M)	F:M = 0.7053:0.2947	F:M = 0.7980:0.2120

## Discussion

Arrhenotokous reproduction is a unique biological process, all unfertilized eggs are haploid and develop as males, while fertilized eggs are diploid and develop as females. This process enables virgin females to survive and produce male offspring, which, in turn, build a bisexual cohort through oedipal mating (mating with their sons)^46^. Arrhenotoky with oedipal mating is not only important to dispersion, colonization and sex allocation^47^, but is also an important aspect in genetics and evolution^48^. It has previously been reported in mites^30,46^, parasitoid wasps^49^, and thrips^50^. Several important pest species, including *F. occidentalis* and the worldwide distributed two-spotted spider mite (*Tetranychus urticae* Koch), are capable of reproducting in this manner. In order to calculate population growth rate (i.e., intrinsic rate of increase and finite rate of increase), it is assumed that the population approaches a stable age-stage distribution. In arrhenotokous reproduction, virgin females produce only male population prior to oedipal mating. Because the population begins bisexual reproduction only after the oedipal mating, it is invalid to calculate the intrinsic rate of increase for the arrhenotokous cohorts based on the fecundity data of male eggs. Tuan *et al*. described methods for calculating the intrinsic rate of increase for arrhenotokous cohorts of two-spotted spider mites^30^.

Using the age-stage, two-sex life table, enabled us to demonstrate the differences in all aspects of population characteristics between the arrhenotokous and bisexual cohorts of a species (Table 1). We determined that when more oedipal mating males were present, a female was capable of producing more female offspring. The 1 F:nM, 1 F: 6 M and 1 F: 5 M sex ratios of oedipal mating cohorts produced higher proportions of female offspring than other arrhenotokous cohorts.

Gaum *et al*.^21^ constructed life tables for *F. occidentalis* reared on English cucumbers, (*Cucumis sativus* L.) cv. ‘Pepinex’, at five temperatures and reported a total immature developmental time of 14.71 days at 25 °C; a time that was longer than that obtained in this study when immatures were fed on purple cabbage^21^. The difference may be attributable to differences in the host plants. The mean fecundity and intrinsic rate of increase reported by Gaum *et al*.^21^ were 9.65 eggs and 0.30 d^−1^, respectively. Akca *et al*. used the following equation to examine the intrinsic rate of increase^42^:

12 $${e}^{-r(a+1)}F\cdot {l}_{a}={\rm{1}}$$

where *F* is the mean fecundity, *l*_*a*_ is the preadult survival rate, and *a* is the age of first offspring. Gaum *et al*.^21^ used Birch’s^25^ method with the following equation to estimate the intrinsic rate of increase:

13 $$\sum _{x=1}^{N}{e}^{-rx}{l}_{x}{m}_{x}={\rm{1}}{\rm{.}}$$

To affirm the intrinsic rate of increase found by Gaum^21^, we, instead, used the following equation:

14 $${e}^{-ra}F\cdot {l}_{a}={\rm{1}}{\rm{.}}$$

By substituting equation 14 for equation 13, and using the values; *a* = 15.71, *F* = 9.65, and *l*_*a*_ = 1, we obtained an *r* value of 0.144 d^−1^. This indicates that even if all 9.65 eggs were laid on the first day of adulthood (i.e., 15.71 d), the intrinsic rate of increase found by Gaum *et al*.^21^ should be less than 0.15 d^−1^. It is clear that the *r* value of 0.30 d^−1^ obtained by Gaum *et al*.^21^ is likely in error. Similar errors were noted in other temperature settings. Zhang *et al*.^22^ reported higher fecundities for *F. occidentalis* at 27 °C, i.e., 49.46, 76.62, 66.44, 24.95 and 7.67 first instar larvae/female when reared on cucumber, cabbage, bean, tomato, and capsicum, respectively after using Birch’s^25^ calculation method. Zhang *et al*. reported the highest intrinsic rate of increase occurred on cucumber (0.208 d^−1^), followed by cabbage (0.184 d^−1^), bean (0.164 d^−1^), tomato (0.100 d^−1^) and capsicum (0.017 d^−1^). These values are acceptable when examined with Equations 12 or 14 ^22^.

Because the production of female offspring in arrhenotokous reproduction must have occurred after oedipal mating, the APOP and TPOP for production of female offspring is significantly longer than it is for male offspring. In addition, the length of the APOP and TPOP needed for production of female offspring decreased with increases in the number of oedipal mating males. The longer APOP and TPOP durations, lower fecundity, and decreased number of oviposition days for production of female offspring in arrhenotokous reproduction are similar to those observed by Tuan *et al*.^30^ in arrhenotokous cohorts of *Tetranychus urticae* Koch (Acari: Tetranychidae). Based on these observations, it can be concluded that, the longer APOP and TPOP durations are likely characteristic of arrhenotokous reproduction.

The female adult longevity in the bisexual cohort was significantly shorter than in females in all of the arrhenotokous cohorts, while female adults not subjected to oedipal mating survived the longest (Table 1). Although no significant differences were found in female adult longevity among the various cohorts with constant numbers of oedipal mating males, female adult longevity in the cohort with continuous oedipal mating (1 F: nM) was significant shorter than that of the cohorts containing constant numbers of oedipal mating males (Table 1). The differences in adult longevities can also be observed in the life expectancy (Fig. 3). The shorter longevity of female adults observed in the two-sex cohort may be due to the energy expenditures involved in bisexual reproduction.

Kumm and Moritz observed that 5 out of 1016 unfertilized eggs of *F. occidentalis* were females, i.e., approximately 0.5%^15^. In the present study, prior to the first female egg being produced after oedipal mating, there were 2189, 2143, 1907, 1586, 1180, 1667, and 2648 male eggs produced by arrhenotokous females in the 1 F: 1 M, 1 F: 2 M, 1 F: 3 M, 1 F: 4 M, 1 F: 5 M, 1 F: 6 M, and 1 F: nM treatments, respectively. In addition, no female offspring emerged from the 4156 eggs produced by the females in the 1 F:0 M treatment. In summary, no female eggs among a total of 17,476 unfertilized eggs were produced during the course of this study.

Arrhenotokous reproduction is a unique feature, which enables a single virgin female to establish a two-sex population through oedipal mating. This phenomenon must inevitably have contributed to the dominance that *F. occidentalis* has attained among economically important phytophagous thrips in many regions worldwide and have been an integral part of their successful invasion and establishment in so many countries. A thorough understanding and accurate description of this unique form of reproduction is critical in predicting and preventing future invasions and outbreaks of this group of pests.

Because traditional female age-specific life tables^23–26^ overlook the males in a population, they are incapable of accurately portraying arrhenotokous reproduction and, consequently, cannot properly be applied in pest management or biological control programs involving this unique reproduction system. In contrast to these female age-specific life tables, the age-stage, two-sex life table has been designed to fully describe the stage differentiation and to include both sexes (Figs 1 and 2). In this paper, we demonstrated that the application of the age-stage, two-sex life table offers a more detailed description of demographic variables and avoids many of the problems inherent to the female age specific life tables as discussed by Huang and Chi^27^. In order to implement a successful economic and sustainable management program for these important pests, it is crucial to take into consideration the effect arrhenotokous reproduction has on the growth rate of the population and on pest management decisions.
